# A Multifunctional Double-Network Hydrogel Based on Bullfrog Skin Collagen: High-Value Utilization of Aquaculture By-Products

**DOI:** 10.3390/foods14111926

**Published:** 2025-05-29

**Authors:** Chunyu Song, Xiaoshan Zheng, Ying Lu

**Affiliations:** 1 College of Food Science and Technology, Shanghai Ocean University, Shanghai 201306, China; gintama8818@gmail.com (C.S.); zxs961023@163.com (X.Z.); 2 Shanghai Engineering Research Center of Aquatic-Product Processing & Preservation, Shanghai 201306, China; 3 National R&D Branch Center for Freshwater Aquatic Products Processing Technology (Shanghai), Shanghai 201306, China

**Keywords:** bullfrog skin collagen, self-healing, antibacterial and hemostatic activity, high-value utilization of aquaculture by-products

## Abstract

Bullfrog skin, as a by-product of bullfrog processing, is an ideal source of high-quality collagen due to its high protein content and low-fat characteristics. However, its comprehensive utilization is relatively low, and the discarded skins cause resource waste and environmental pollution. In this study, a citric acid extraction process for frog skin collagen was established through single-factor optimization. A multifunctional double-network hydrogel was developed by combining the prepared high-purity type I collagen with oxidized hyaluronic acid (OHA). Due to the network structure design of Schiff base bonds and Zn^2+^ coordination bonds, the mechanical strength of the hydrogel based on collagen and OHA compositing Zn^2+^ (Gel–CO@Zn) enhanced significantly. It was found that the Gel–CO@Zn hydrogel had strong tissue adhesion (16.58 kPa shear strength), rapid self-healing (<6 h), and low hemolysis (<5%). Furthermore, the Gel–CO@Zn hydrogel could reduce the survival rate of *Staphylococcus aureus* and *Escherichia coli* to 1.06% and 6.73%, respectively, showing good antibacterial properties. Through the treatment of Gel–CO@Zn, the clotting time was shortened from 433 s to 160 s and greatly reduced the blood loss (>60%) in the liver injury model of male Kunming mice. This research not only presents a novel approach for the high-value utilization of aquaculture by-products but also establishes a new paradigm for developing cost-effective, multifunctional biomedical materials, demonstrating the transformation of waste into high-value resources.

## 1. Introduction

Bullfrogs are increasingly recognized as a high-quality collagen source due to their high protein content and low fat [1]. In 2023, China’s bullfrog farming industry achieved a production volume of 1 million tons, generating an economic value of approximately USD 4.23 billion [2]. This growing consumption has generated a large quantity of bullfrog skin as a by-product. Since most bullfrog skin is discarded as waste, this leads to resource waste and environmental pollution [3]. Bullfrog skin, rich in type I collagen, has potential biomedical applications [4]. Wang et al. [5] repurposed discarded bullfrog skin and fish scales as biomaterials to develop a multiscale hybrid functional porous “waste-to-resource” bone graft material. The extraction and reaction pathways employed in scaffold fabrication demonstrated environmental sustainability. This biocompatible structure effectively supported the proliferation and maturation of human bone progenitor cells. This study revealed that collagen derived from farmed bullfrogs, serving as an aquatic collagen source, does not elicit any detectable inflammatory responses or immunogenic reactions, demonstrating promising biocompatibility for biomedical applications. Extracting collagen from aquatic products offers dual advantages in both economic and environmental aspects. Collagen derived from aquatic sources demonstrates numerous benefits including good biocompatibility, superior absorption characteristics, and fewer religious and regulatory restrictions compared to conventional sources. At present, there have been many studies on the high-value utilization of collagen from by-products of aquatic products. For example, Govindharaj et al. [6] use sustainably utilized discarded eel skins to fabricate 3D scaffolds for tissue engineering applications. Ma et al. [7] designed a novel bioactive gelatin hydrogel-loading peptide derived from Atlantic cod (*Gadus morhua*).

Natural collagen-based hydrogels have emerged as advantageous options in both hard and soft tissue restoration by functioning as biological frameworks that support cellular proliferation [8]. Though natural collagen-based hydrogels offer promising benefits for tissue regeneration, their clinical utility faces serious constraints due to poor mechanical flexibility and swift breakdown by enzymes. To address these limitations, researchers have implemented various modification strategies involving external chemical, physical, or biological crosslinking agents to alter collagen’s molecular architecture, thereby slowing degradation rates and strengthening structural integrity [9]. For example, Menezes et al. [10] prepared a hydrogel based on collagen extraction from Nile tilapia skin (*Oreochromis niloticus*) and hyaluronic acid, which exhibited good rheological properties and swelling. However, this single-network hydrogel still has functional limitations. The development of double-network (DN) hydrogels, formed by combining two distinct polymer systems, has gained significant traction recently due to their unique structural, physicochemical, and functional properties. These DN structures offer a wider spectrum of properties compared to the single network (SN), expanding their potential applications in various fields, especially in biomaterials [11]. For example, Kang et al. [12] designed a biological macromolecule hydrogel based on recombinant type I collagen/chitosan scaffold. The hydrogel could accelerate full-thickness healing of skin wounds. Chen et al. formulated dual-crosslinked hydrogels using robust fish gelatin, transglutaminase, and carrageenan, which resulted in enhanced mechanical properties and improved heat resistance following the double crosslinking process [13]. Carrageenan-only crosslinked hydrogels suffered from irreversible plastic deformation under stress, highlighting the need for synergistic covalent/non-covalent strategies to achieve balanced toughness and elasticity.

Hyaluronic acid (HA), a natural glycosaminoglycan commonly found in connective tissues, has been shown to regulate cell migration and facilitate bone tissue regeneration [14]. Higher molecular weight HA-based hydrogels tend to have higher viscosity and slower degradation rates [15]. Yang et al. [16] illustrated the remarkable capabilities of injectable hydrogels derived from HA for wound treatment applications, highlighting their superior adhesion to wound surfaces and capacity to adapt and fill irregularly shaped wound cavities. Liu et al. [17] discovered that the simultaneous application of mesenchymal stem cell-derived exosomes with HA gel substantially decreased oxidative stress levels in cartilage cells. However, native HA exhibits significant susceptibility to enzymatic degradation in physiological environments and demonstrates less mechanical stability, thereby limiting its applicability in load-bearing biomedical applications. By introducing aldehyde groups to HA through peroxidation reactions to generate OHA, the researchers not only enhanced the resistance of HA to hyaluronidase but also achieved dynamic covalent crosslinking with ε-amino-rich polymer through pH-responsive Schiff base bonds. For example, Yu et al. [18] designed a multifunctional hydrogel by Schiff base reaction between carboxymethyl chitosan (CMCS) and OHA. This innovative hydrogel demonstrated remarkable shape memory capabilities and self-healing characteristics, while also exhibiting compatibility with 3D printing technologies. Tan et al. [19] developed injectable composite hydrogels containing hydroxyapatite through Schiff base reactions between OHA and CMCS, creating materials that effectively supported cell encapsulation and showed promising applications for bone defect restoration. These studies indicate that OHA is an excellent polymer material with the potential to prepare multifunctional hydrogels.

**Scheme 1:**
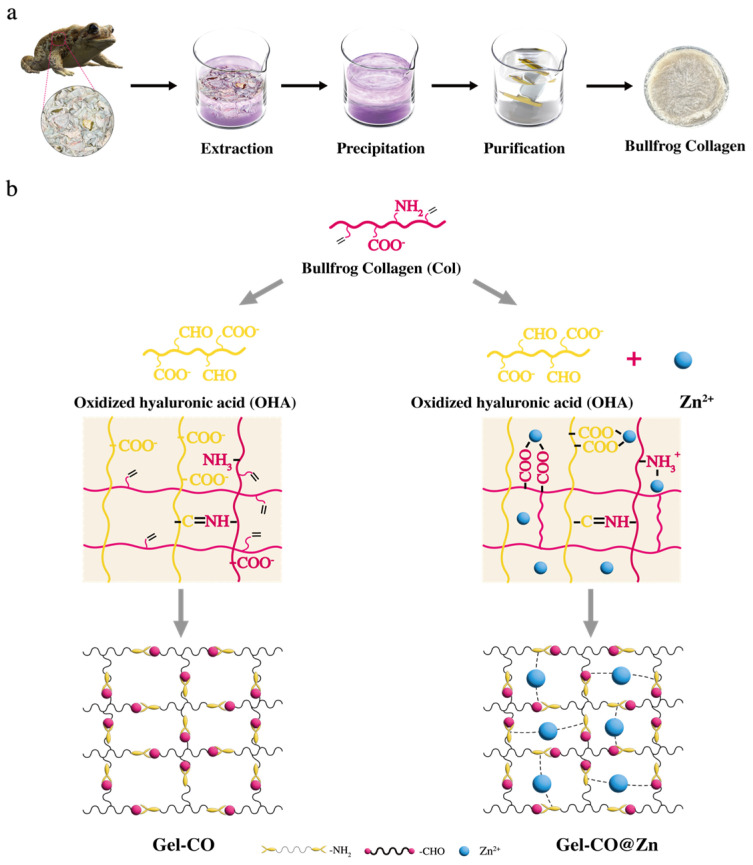
(**a**) Preparation of bullfrog collagen. (**b**) Preparation of Gel–CO/Gel–CO@Zn hydrogels.The pink line represents Col, and the yellow line represents OHA.

Although the processing of by-products of Marine and freshwater fish as sources of collagen has been widely studied, bullfrog skins are rich in high-quality collagen and have not yet been developed and utilized. In order to promote the comprehensive utilization of bullfrog skins, in this study, the extraction process of bullfrog skin collagen was optimized through single-factor experiments. A novel double-network multifunctional composite hydrogel based on collagen and OHA (Gel–CO) was prepared through a Schiff base reaction. As illustrated in Scheme 1, the aldehyde group of OHA interacted with numerous efficacious amino groups present on the collagen (Col) side chain, resulting in the formation of dynamic imine bonds. The innovative aspect of our design lies in the incorporation of Zn^2+^ ions into the Gel–CO system as a second crosslink via metal coordination bonds to form Gel–CO@Zn. The embedding of Zn^2+^ into the Col-OHA system promoted the formation of metal coordination bonds, and this dual-network approach not only introduced antimicrobial properties but also strengthened the dynamic network.

## 2. Materials and Methods

### 2.1. Materials

Bullfrog (*Rana catesbeiana*) skin tissues were sourced from a commercial food processing company in China. Citric acid (AR, 99.5%) was purchased from Rhawn Reagent (Shanghai, China). Sodium chloride was purchased from Sangon Biotech (Shanghai, China). *Escherichia coli* (ATCC25922) and *Staphylococcus aureus* (CMCC26003) were obtained from Shanghai Luwei Technology Co., Ltd. (Shanghai, China). Hyaluronic acid (97%) (average MW 400,000–700,000) was from Macklin (Shanghai, China). Analytical grade reagents, including sodium periodate (NaIO_4_), zinc chloride (ZnCl_2_), phosphate-buffered saline (PBS, pH 7.4), and ethylene glycol were procured from Sigma-Aldrich (St. Louis, MO, USA). All chemical reagents were of analytical grade and used as received. Male mice, weighing 50–60 g, were obtained from JieSiJie Laboratory Animal Co., Ltd. (Shanghai, China) and individually housed with ad libitum access to food and water.

### 2.2. Optimization of Collagen Extraction from Bullfrog Skin

Fresh bullfrog skin fragments (0.5 × 0.5 cm^2^) were mixed with 0.1 M NaOH (1:10 *w*/*v*) at 4 °C for 24 h. After washing with ultrapure water until the solution achieved a neutral pH. The pretreated bullfrog skin was subjected to extraction optimization according to the following single-factor experimental methods.

#### 2.2.1. Effect of Acid Concentration on Collagen Extraction Yield

Ten grams of bullfrog skin were treated for 24 h at a bullfrog skin–citric acid solution ratio of 1:40 using citric acid solutions with concentrations of 0.3, 0.6, 0.9, 1.2, and 1.5 mol/L to determine the optimal acid concentration for collagen extraction.

#### 2.2.2. Effect of Bullfrog Skin–Citric Acid Solution Ratio on Collagen Extraction Yield

The 10 g of bullfrog skin was treated with 0.3 mol/L citric acid solution for 24 h at solid–liquid ratios of 1:20, 1:40, 1:60, 1:80, and 1:100 (*w*/*v*) to evaluate the effect on collagen extraction yield.

#### 2.2.3. Effect of Extraction Time on Collagen Extraction Yield

Ten grams of bullfrog skin were subjected to extraction with 0.2 mol/L citric acid solution at a bullfrog skin–citric acid solution ratio of 1:20 for 12, 24, 36, 48, and 60 h to assess the impact of extraction duration on collagen yield.

The experiments were conducted using the optimal conditions determined from the above single-factor experiments, and the collagen extraction rate was calculated accordingly. In brief, fresh bullfrog skin fragments (0.5 × 0.5 cm^2^) were mixed with 0.1 M NaOH (1:10, *w*/*v*) at 4 °C for 24 h. After washing with ultrapure water until the pH of the solution was neutral, the pretreated skin fragment (100 g) was mixed with 0.6 M citric acid at a ratio of 1:40 (*w*/*v*) under continuous stirring at 4 °C for 24 h. Subsequently, 2.6 M NaCl was added and the reaction was maintained for 2 h. The precipitate was collected by centrifugation (10,275× *g*) at 4 °C for 30 min and resuspended in ultrapure water. The suspension was dialyzed (MWCO 15 kDa) against ultrapure water for 72 h with regular buffer changes at 8 h intervals. The purified collagen was lyophilized and stored at −20 °C for subsequent analysis, according to the following formula for collagen extraction yield:

$$Collagen\;extraction\;yield\left(\%\right)=\frac{Collagen\;content\;in\;the\;extract}{Original\;collagen\;content}\times 100\%$$

The original collagen content in bullfrog skin was determined by converting from the hydroxyproline content measured in the skin samples.

### 2.3. Synthesis and Quantification of OHA

OHA was prepared by the method of Xu et al. [20], with slight modifications. Briefly, sodium hyaluronate (2.0 g) was dissolved in ultrapure water (150 mL), followed by the addition and thorough mixing of sodium periodate solution (0.5 mol/mL, 10 mL). The oxidation reaction was conducted in darkness at room temperature for 24 h and then terminated using 5 mL of ethylene glycol. The mixture was dialyzed (MWCO 3.5 kDa) against ultrapure water (20 volumes, 2 L) with water replaced every 8 h for 72 h and freeze-dried. The purified OHA was stored at 4 °C. Aldehyde content was quantified using hydroxylamine hydrochloride titration revealing a 5.6% degree of oxidation.

### 2.4. Preparation of Composite Hydrogels (Gel–CO and Gel–CO@Zn)

OHA-Zn^2+^ was prepared by dissolving OHA in 1% ZnCl_2_ solution (10%, *w*/*v*). Then, 100 μL of OHA (10%, *w*/*v*) in 0.1 M phosphate-buffered solution (PBS, pH = 5.0) or 100 μL of OHA-Zn^2+^ was mixed with 1.0 mL Col solution under stirring; the total concentration of Col in the mixed system was fixed to be 10% (*w*/*w*). Gel–CO and Gel–CO@Zn were obtained by pouring the mixture into a mold (5 mm height × 10 mm diameter) and equilibrated at 25 °C for 2 h.

### 2.5. Physicochemical Characterization of Gel–CO and Gel–CO@Zn

The dual-network hydrogel system (Gel–CO@Zn) was designed based on established principles of dynamic crosslinking chemistry. The concentrations and ratios used in the hydrogel synthesis were determined based on preliminary optimization experiments and the existing literature on Schiff-based hydrogels [21]. The infrared spectra of Col, HA, and OHA were analyzed using the method reported by Ma et al. [22]. The morphology of hydrogels was observed using the method already reported by Cheng et al. [23]. For SEM imaging, hydrogel samples were flash-frozen in liquid nitrogen followed by lyophilization to maintain the original structure. A thin gold layer was deposited on the freeze-dried samples by sputter coating under a vacuum for 60 s for conductivity enhancement.

### 2.6. Swelling and Degradation Analysis of Gel–CO and Gel–CO@Zn

To assess the water uptake capacity, pre-weighed Gel–CO and Gel–CO@Zn samples were immersed in 10 mmol/L PBS buffer (pH 7.4) at 37 °C, respectively. At predetermined time intervals (15 min, 1 h, 2 h, 4 h, and 8 h), the samples were removed and weighed from the media to remove the additional surface media. The wet and dried weight of samples was marked as *W_s_* and *W_0_*, respectively. The swelling ratio (SR) was calculated as follows:

$$Swelling\;ratio\left(\%\right)=\frac{W_{s}-W_{o}}{W_{o}}\times 100\%$$

The decomposition of biological materials represents a fundamental process. For in vitro degradation studies, pre-weighed lyophilized Gel–CO and Gel–CO@Zn were immersed in collagenase–PBS solution (0.02 U/mL) at 37 °C, and incubated under continuous orbital shaking at 120 rpm. The enzymatic solution was refreshed daily to maintain constant enzyme activity. At predetermined time intervals (4 h and 8 h), the samples were thoroughly rinsed with deionized water, lyophilized, and weighed. The initial weight and weight after degradation were marked as *W_0_* and *W_t_*, respectively. The degradation rate was calculated as follows:

$$Degradation\;ratio\left(\%\right)=\frac{W_{o}-W_{t}}{W_{o}}\times 100\%$$

### 2.7. Analysis of Rheological Property

The rheological properties of the GelCO and GelCO@Zn hydrogels were estimated at 25 °C using a rheometer (HR-2, Discovery Series Hybrid Rheometer, TA Instruments, New Castle, DE, USA). The measurements of storage modulus (G’) and loss modulus (G”) were carried out at a frequency of 1 Hz and a dynamic strain of 1%. The steady shear test was utilized to characterize the shear thinning behavior of the hydrogels and identified by measuring the viscosity in relation to the shear rate.

### 2.8. Self-Healing and Adhesiveness Evaluation

To investigate the self-healing capability of Gel–CO@Zn, the material was first cut in the middle using a surgical blade. One half of the sample was stained with malachite green, and the two pieces were then brought into contact and reassembled. The reconnected sample was placed in a glass container to maintain a moist environment. The experiment was conducted at 25 °C, and the self-healing process was monitored over time. Corresponding photographs and microscopic images were captured at 5 s and 6 h.

For the lap shear test, a hydrogel sample (20 × 20 mm^2^) was uniformly adhered to porcine skin (20 × 20 mm^2^). After stabilization for 1 h, the shear strength of hydrogel was analyzed using a force–displacement curve. Similarly, for the shear strength measurement, samples (20 × 20 mm^2^) were prepared and adhered to porcine skin (20 × 20 mm^2^). The shear strength was calculated by dividing the maximum force by the adhesion area. All measurements (*n* = 3) were performed at a constant tensile speed of 50 mm/min.

### 2.9. In Vitro Antimicrobial Analysis

The antibacterial activity was determined according to the method reported by Chen et al. [24] with some modifications. The Gel–CO and Gel–CO@Zn samples were sterilized under ultraviolet (UV) irradiation for 2 h in 24-well plates using a clean bench (Model: SW-CJ-1F) equipped with a 30 W UV lamp providing an intensity of 107 μW/cm^2^ (1.07 × 10^−1^ mW/cm^2^). *Escherichia coli* (ATCC25922) and *Staphylococcus aureus* (CMCC26003) diluted in sterile PBS were used as the control group (1 × 10^5^ CFU/mL). Sterilized hydrogel samples were added into aliquots of bacterial suspension (1 × 10^5^ CFU/mL) and used as test group. After incubation on a shaker at 37 °C for 12 h, the bacterial suspensions of control group were diluted to 1 × 10^6^ CFU/mL with PBS buffer (pH 7.4), and 20 μL bacterial suspension of each group was plated onto LB agar plates and incubated at 37 °C for 18 h to assess the growth status of bacteria. Subsequently, bacterial suspension was plated onto LB agar plates and incubated at 37 °C for 18 h. Colony-forming units (CFUs) were counted and photographed to assess bacterial viability. Antibacterial efficacy was calculated using the following equation:

$$Antibacterial\;viability(\%)=\frac{{CFU}_{hydrogel}}{{CFU}_{control}}\times 100\%$$

where CFU_hydrogel_ represents the number of colonies from hydrogel-treated samples, and CFU_control_ represents the number of colonies from the control group.

### 2.10. Blood Compatibility and Hemostatic Properties

#### 2.10.1. Hemocompatibility of Hydrogels

Male SPF-grade Kunming mice were purchased from Shanghai JieSiJie Laboratory Animal Co. Ltd. (Animal License No. SCXK 2018-0004, Shanghai, China). In this study, all animal experiments and procedures were approved by the Laboratory Animal Ethics Committee of Shanghai Ocean University (SHOU-DW-2021-003) and performed in accordance with the guidelines and regulations of the National Research Council’s Guide for the Care and Use of Laboratory Animals (IACUC No. 3590). Fresh whole blood was collected from a healthy Kunming mouse (male, 7–8 weeks) and immediately anticoagulated with 0.3% (*w*/*v*) trisodium citrate dihydrate. The blood samples were used within 24 h of collection to maintain freshness. The collected blood was centrifuged at 924× *g* for 10 min to isolate red blood cells (RBCs). The RBCs were washed with PBS buffer until the supernatant became clear and colorless. The purified RBCs were then diluted with PBS to create a 2% (*v*/*v*) RBC suspension. All experiments were performed in triplicate (*n* = 3).

The hemolysis ratios of samples were tested using a modified version of a previously reported method [25]. To evaluate blood compatibility, Col, Gel–CO, and Gel–CO@Zn hydrogel samples were each mixed with 200 μL of 2% RBC suspension and 800 μL of PBS, and then incubated at 37 °C for 1 h. Positive controls (200 μL of RBC suspension + 800 μL of deionized water) and negative controls (200 μL of RBC suspension + 800 μL of PBS) were prepared in parallel. After incubation, all samples were centrifuged at 5000 rpm for 5 min, and the supernatant absorbance was measured at 540 nm. The hemolysis rate was calculated using the following formula:

$$Hemolysis\;ratio\left(\%\right)=\frac{{OD}_{sample}-{OD}_{PBS}}{{OD}_{water}-{OD}_{PBS}}\times 100\%$$

#### 2.10.2. Blood Clotting Assessment

For determining whole blood clotting time, 200 μL of mouse blood was placed in a 2 mL centrifuge tube and pre-warmed to 37 °C. The blood was then recalcified by the addition of 10 mM CaCl_2_. Subsequently, the blood was mixed with a 600 μL sample and incubated at 37 °C. The clotting time referred to the time when the blood did not flow if the tube was inverted. Recalcified blood untreated with hydrogels was used as the control. Each experiment was conducted in triplicate (*n* = 4).

The blood clotting index (BCI) was determined according to previously reported methods [26]. Briefly, blood coagulation was activated by adding 50 μL of 0.1 mM CaCl_2_ to 500 μL of anticoagulated whole blood. A 10 μL aliquot was then evenly distributed onto the hydrogel surface pre-warmed to 37 °C. After incubation for 10 min, 1 mL of ultrapure water was gently added to lyse unclotted RBCs. The absorbance of 200 μL of supernatant was measured at 545 nm (A_sample_). A blank control was established by mixing 10 μL of blood with 1 mL of ultrapure water (A_blank_), and the BCI was calculated as follows (*n* = 3):

$$Blood\;clotting\;index\left(\%\right)=\frac{A_{sample}}{A_{blank}}\times 100\%$$

#### 2.10.3. In Vivo Hemostasis Evaluation

Animal hemostasis experiments for mice were performed according to the method modified by Qiao et al. [27]. A total of 40 male Kunming mice (50–60 g) were used in this study. The mice were randomly divided into four groups (*n* = 10), including the control group, Gel–CO, Gel–CO@Zn, and liquid bandage. Within each group, 5 mice were used for the liver injury model and 5 mice for the tail amputation model. The hemostatic efficacy of Gel–CO/Gel–CO@Zn hydrogel was evaluated using mice liver injury and tail amputation models to simulate visceral and dismembered hemorrhage scenarios in humans [28]. Male Kunming mice (50–60 g) were anesthetized with isoflurane and euthanized after the experiments. For the liver injury model, the liver was exposed through an abdominal incision and the surrounding tissue fluid was removed. Pre-weighed filter paper was placed underneath the liver, and either filter paper, liquid bandage, or size-fixed hydrogels were immediately applied to the bleeding site. A control group received no treatment after liver injury. In the tail amputation model, one-third of the tail was cut and exposed for 15 s to allow initial blood loss. The wound was then treated with 200.0 μL of hydrogel, commercial liquid bandage, or left untreated. Then, the blood loss and hemostasis time were monitored after the bleeding stopped.

### 2.11. Statistical Analysis

All experiments were conducted with three or four independent biological replicates as specified in figure legends, each containing triplicate technical measurements. And data were presented as mean ± standard deviation (SD). Data were analyzed with SPSS 26 software (IBM Corporation, Armonk, NY, USA). Statistical comparisons between paired groups were performed using Student’s *t*-test, while multiple group comparisons were analyzed using one-way analysis of variance (ANOVA) followed by Tukey’s post hoc test. ANOVA with Tukey’s test was used to analyze the difference between samples (*p* < 0.05, significant differences), with significance levels indicated as * *p* < 0.05, ** *p* < 0.01, *** *p* < 0.001, and **** *p* < 0.0001. All statistical analyses were conducted using GraphPad Prism (Version 7.0; GraphPad Software, Inc., La Jolla, CA, USA) or R (Version 4.0.3).

## 3. Results

### 3.1. Optimization of the Extraction Condition of Collagen from Bullfrog Skin

**Figure 1:**
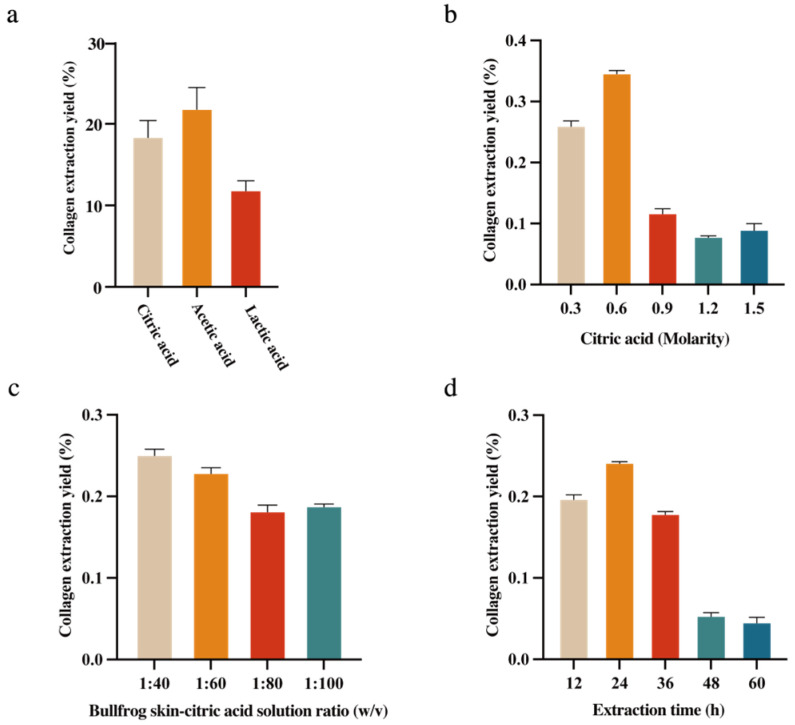
(**a**) Effects of types of acids; (**b**) extraction rate of collagen from bullfrog skin; (**c**) extraction time; and (**d**) bullfrog skin–citric acid solution ratio, the vertical bars represent the standard errors (*n* = 3).

The extraction conditions play a key role in influencing the yield and physicochemical properties of collagen [29]. Different acids affect collagen extraction rates. Citric acid, acetic acid, and lactic acid were used in this study to extract collagen from bullfrog skin. Figure 1a shows that collagen extraction using lactic acid was significantly lower than with citric acid and acetic acid. The extraction rates of citric acid and acetic acid were similar, both exceeding 18%. However, acetic acid has a strong irritating odor, which is unfavorable for experimental operations and subsequent product development. In contrast, citric acid produces no odor during operation. Therefore, citric acid was selected as the experimental acid for collagen extraction. Figure 1b shows that different citric acid concentrations lead to different collagen extraction yields. When the citric acid concentration is below 0.6 M, the collagen extraction rate increases, reaching its maximum value at 0.6 M, where the extraction rate exceeds 30%. As the citric acid concentration continues to increase, the extraction rate actually decreases. This may be because higher concentrations damage the collagen structure, causing denaturation of the collagen. As shown in Figure 1c, with the increase in the bullfrog skin–citric acid solution ratio, the collagen extraction rate first rises and then falls. The collagen extraction yield reaches its highest at a bullfrog skin–citric acid solution ratio of 1:40. Further increasing the bullfrog skin–citric acid solution ratio not only reduces the extraction rate but also wastes more reagents. Therefore, 1:40 was selected as the optimal ratio. As shown in Figure 1d, between 12 and 24 h, the collagen extraction rate continuously increases, reaching its peak extraction rate at 24 h. After 24 h, it begins to show a declining trend, with the extraction rate falling below 10% at 48 h. Insufficient collagen extraction time can result in incomplete extraction of collagen, while extraction time that is too long can lead to the breakdown of collagen because the acid can also damage the collagen structure. Based on the results of the above experiments, using citric acid as the experimental acid, with a bullfrog skin–citric acid solution ratio of 1:40 and 0.6 M citric acid for an extraction period of 24 h, the final collagen extraction rate was calculated to be 36.28%.

### 3.2. Characterization of Col and OHA

**Figure 2:**
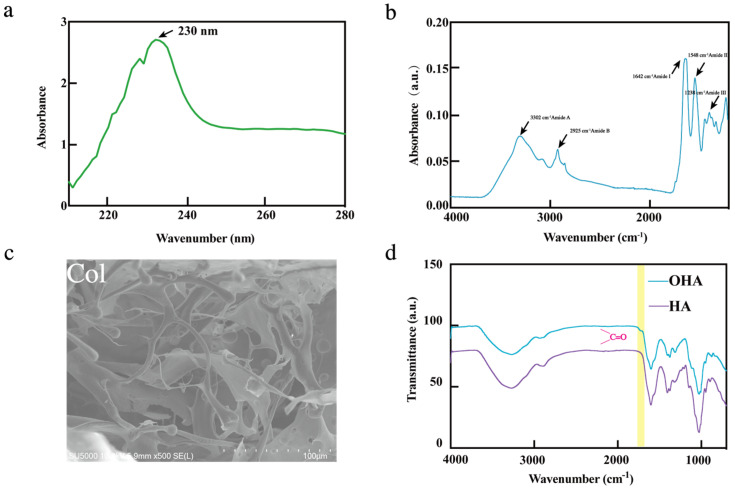
Characterization of Col, OHA; (**a**) UV spectra of Col; (**b**) FT-IR spectrum of Col; (**c**) SEM images of Col, scale bar = 200 µm; and (**d**) FT-IR spectrum of HA and OHA analysis.

In order to verify the purity and confirm the type of collagen, the extracted collagen was first analyzed by UV spectra and FTIR. As shown in Figure 1a, the spectroscopic analysis revealed that the obtained Col exhibited maximum absorption around 230 nm, which is attributable to the presence of chromophores including CONH_2_, -COOH, and C=O functional groups within its polypeptide backbone structure [30]. The result was in agreement with the reports on type I collagens from the skin of Nile tilapia (225 nm) [31], purple-spotted Bigeye Snapper (230 nm) [32], and channel catfish (232 nm) [33], indicating that the collagen extracted from bullfrog skin corresponds to the characteristic UV spectral features of type I collagen. The extracted collagen was further analyzed by FTIR to verify its purity and structural integrity, such as whether there is a characteristic peak of type I collagen. Apparently, FTIR results showed that the collagen exhibits N-H stretching vibration at 3302 cm^−1^ in the amide A region, and asymmetric stretching vibration of methylene groups at 2925 cm^−1^ in the amide B region. Moreover, there was C=O stretching vibration at 1642 cm^−1^ in the amide I region, N-H bending at 1548 cm^−1^ of the amide II region, and C-N stretching vibrations at 1238 cm^−1^ in the amide III region (Figure 2b). These results were consistent with FTIR spectra of collagen with helical structures from different frog species (*R. nigromaculata* and *Rana tigerina*) [34,35]. These spectral characteristics confirmed the existence of the triple helical structure in bullfrog collagen [36]. The SEM was used to evaluate the structural integrity of the collagen. Figure 2c exhibited a hierarchical architecture composed of irregularly arranged fibrillar networks with multi-layered aggregation, suggesting that the bullfrog skin-derived collagen retained its native fibrillar integrity, making it potentially suitable for biomedical applications such as wound dressings, hemostatic agents, and cell culture matrices. In addition, FTIR was used for the identification and analysis of OHA. Compared to the FTIR spectra of HA, it was found that OHA had a new peak at 1726 cm^−1^ (Figure 2d), which was caused by the stretching vibration of the -C=O- bond in the aldehyde group [37]. The FTIR analysis result suggested that the HA was successfully transformed into OHA.

### 3.3. Preparation and Characterization of Gel–CO and Gel–CO@Zn Hydrogels

**Figure 3:**
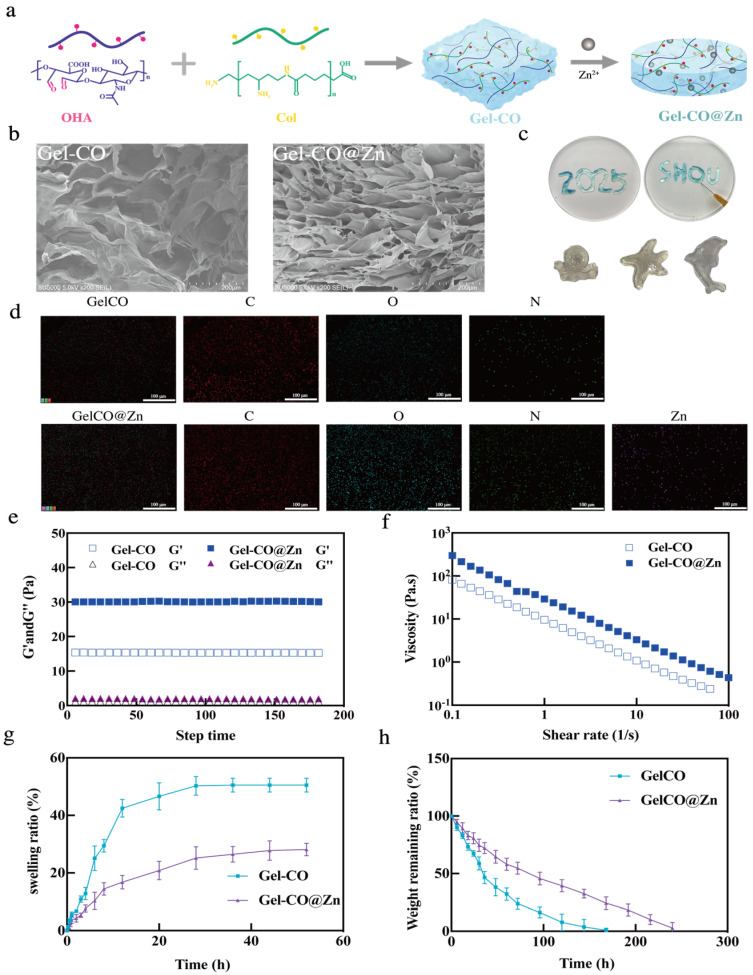
Characterization of Gel–CO and Gel–CO@Zn hydrogels; (**a**) graphical reaction of hydrogels; (**b**) SEM images of Col, Gel–CO, and Gel–CO@Zn; (**c**) the injectability and moldability of Gel–CO@Zn; (**d**) EDS analysis of Gel–CO and Gel–CO@Zn; (**e**) time sweep experiments of Gel–CO and Gel–CO@Zn; (**f**) the steady shear-thinning properties of Gel–CO and Gel–CO@Zn, scale bar = 200 µm; (**g**) in vitro swelling of Gel–CO and Gel–CO@Zn; and (**h**) in vitro degradation of Gel–CO and Gel–CO@Zn (in PBS with pH 7.4 at 37 °C), the vertical bars represent the standard errors (*n* = 3).

Gel–CO and Gel–CO@Zn hydrogels were prepared by the reaction between Col and OHA via Schiff base reaction, and coordinated with Zn^2+^ (Figure 3a). The morphology of Gel–CO and Gel–CO@Zn is shown in Figure 3b, and it was observed that both hydrogels exhibited three-dimensional and highly interconnected porous structures. The porous walls of Gel–CO were a thin, undulating, and interconnected network due to the Schiff base reaction between collagen and oxidized hyaluronic acid. In contrast, due to the coordination of Zn^2+^, the pore wall of the Gel–CO@Zn hydrogel was thicker and showed a more organized and compact network structure. Figure 3d shows the uniform distribution of Zn^2+^ (purple color) on the surface of Gel–CO. This homogeneous dispersion structure enables efficient and uniform release of Zn^2+^, facilitating controlled and sustained therapeutic delivery. The injectability and moldability of Gel–CO@Zn hydrogel were examined and the results are shown in Figure 3c. Obviously, the hydrogel could be continuously extruded through a 0.5 × 20 mm needle, and be able to write words such as “SHOU” and “2025” or draw images like ink. Moreover, no collapse or fracture phenomenon was observed after a period of time. Consequently, the Gel–CO@Zn hydrogel developed in this study might be used as a potential material for 3D printing.

In addition, the mechanical properties of two hydrogels were analyzed through rheological measurements at 25 °C. Distinct gelation kinetics between Gel–CO and Gel–CO@Zn were explored by time sweep measurements (Figure 3e). It was found that upon zinc ion incorporation, the storage modulus (G’) increased substantially from 15 Pa to 30 Pa, while the loss modulus (G”) remained relatively constant at approximately 1~2 Pa. The mechanical properties of Gel–CO@Zn hydrogel significantly improved due to the dual-dynamic-bond crosslinking based on Schiff base and Zn^2+^ coordination. The viscosity of Gel–CO and Gel–CO@Zn hydrogels at 37 °C was further analyzed. An increase in the shear rate was found to decrease the viscosity of the hydrogels (Figure 3f). The reason might be that shearing breaks the dynamic hydrogel network of hydrogels. It was found that thin and loose structures caused by shear could enhance the self-healing and injection performance of hydrogel [38].

The physical characteristics of hydrogels were investigated by swelling and degradation analysis. Excessive swelling of adhesive hydrogels poses a risk of tissue compression injury in confined spaces, such as the spinal cord [39]. Therefore, it is necessary to control the swelling property of hemostatic hydrogels to maintain certain adhesion strength and avoid adverse compression damage to the surrounding tissues [40]. It was found that Gel–CO expanded significantly within 24 h and reached equilibrium at 28 h, with a swelling ratio of 50.30 ± 3.27% (Figure 3g). However, the swelling of Gel–CO@Zn reached equilibrium at 48 h, and the swelling ratio was 28.13 ± 2.17%, which was only about 1/2 of that of Gel–CO hydrogel. The reason for the lower swelling ratio of Gel–CO@Zn was that the addition of Zn^2+^ increased the crosslinking density of the network structure.

The degradation of two hydrogels was also estimated. As shown in Figure 3h, the Gel–CO and Gel–CO@Zn hydrogels degraded gradually after 160 h and 240 h, respectively. The metal-coordination crosslinks created a more compact architecture, simultaneously restricting water molecule infiltration and enzyme accessibility to cleavage sites. The dense and uniform crosslinking of Gel–CO@Zn reduces its swelling behavior and ensures appropriate degradation kinetics. These characteristics are advantageous for increasing blood viscosity, promoting platelet aggregation and activation, which are critical for hemostasis, and preserving its stability during tissue regeneration.

### 3.4. Self-Healing and Tissue Adhesiveness Properties of Gel–CO@Zn Hydrogel

**Figure 4:**
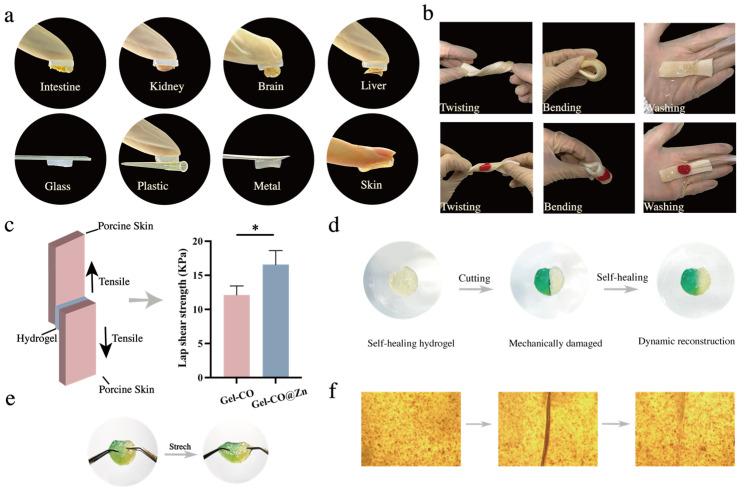
Bio-adhesion and self-healing properties: (**a**) photographs of tissue adhesion situations of Gel–CO@Zn on 4 different organs of mice (intestine, kidney, brain, and liver), glass, plastic, metal, and skin; (**b**) photographs of the blood gel derived from mice and Gel–CO@Zn adhered on wet porcine skin, with standing twists, and washing hydrogel with water; (**c**) schematic of the tensile adhesion test and lap-shear tests of the Gel–CO and Gel–CO@Zn hydrogels; the vertical bars represent the standard errors (*n* = 3); (**d**) self-healing behavior of the Gel–CO@Zn hydrogel; (**e**) photograph of stretching Gel–CO@Zn after healing; and (**f**) microscopic photographs of healing processes (* *p* < 0.05).

Hydrogel adhesion is critical for effective application in biomedical contexts, particularly for hemostatic materials that must maintain stable contact with tissues during bleeding. Strong bio-adhesion ensures the hydrogel remains firmly attached to target tissues under various physiological conditions, including movement, fluid exposure, and mechanical stress. To comprehensively evaluate these essential adhesive properties, we conducted a series of tests examining the performance of our hydrogels across multiple surfaces and conditions. As shown in Figure 4a, Gel–CO@Zn showed good adhesion properties; it could directly adhere to various organs and tissues, such as the intestine, kidney, brain, and liver, as well as to multiple synthetic materials, such as glass, plastic, metal, and skin surfaces without surface modification. Consequently, Gel–CO@Zn had excellent adhesion and broad-spectrum adhesion capabilities, indicating potential as a multifunctional tissue adhesive for biomedical applications [41,42]. In addition, whether the porcine skin is twisted or folded, or rinsed with running water, Gel–CO@Zn could be firmly adhered to the surface and did not fall off even in the presence of blood (Figure 4b). These results not only demonstrated its potential utility in hemorrhagic conditions but also addressed a key limitation of the loss of effectiveness of traditional hydrogels in aqueous environments [43].

Because of the mechanical similarity between porcine skin and human skin tissue [44], we used porcine skin as a model substrate to assess the tissue adhesion performance of two hydrogels. The lap shear strength results are shown in Figure 4c; obviously, Gel–CO@Zn had significantly higher adhesion strength (16.58 ± 2.05 kPa) compared to Gel–CO (12.12 ± 1.33 kPa). These results indicated that the Gel–CO@Zn hydrogel had good adhesion and conformability, and could be used as a potential material for various bleeding conditions.

As another critical property of soft materials, self-healing ability is essential for prolonging their lifespan and improving material reliability. The damage to conventional hydrogel structure is usually irreversible, and the small defects will gradually increase to form larger cracks, resulting in the loss of material properties [45]. The self-healing properties of hydrogels not only improve the defect of the structure of the hydrogel not recovering spontaneously after damage but also promote the development of multifunctional hydrogels and expand their application in biomedicine [46]. Multiple characterization methods were employed to conduct a comprehensive analysis of the self-healing properties of two hydrogels. Firstly, malachite green was utilized as a staining agent to visualize the self-healing behavior at the macroscopic level (Figure 4d). Two freshly cut surfaces were found to heal spontaneously when brought into contact without external intervention, and cracks disappeared after 6 h. Next, when the healed hydrogels were cut again, it was found to heal again and be stretchable (Figure 4e). This is attributed to the fact that the dynamic bonds in hydrogels (Schiff base bonds, Zn^2+^ coordination bonds, and multiple hydrogen bonds) are reversible, thus giving Gel–CO@Zn excellent self-healing properties. This self-healing process of Gel–CO@Zn was validated by optical microscope analysis (Figure 4f). It was observed that the cut cylindrical hydrogels were spliced back together, and the crack almost disappeared after 6 h. The findings confirmed that the restoration mechanism was primarily driven by dynamic covalent bond formation, which is distinct from conventional surface adhesion phenomena.

### 3.5. Evaluation of Antibacterial and Hemostatic Effects

#### 3.5.1. In Vitro Evaluation of Hemocompatibility and Hemostatic Properties

**Figure 5:**
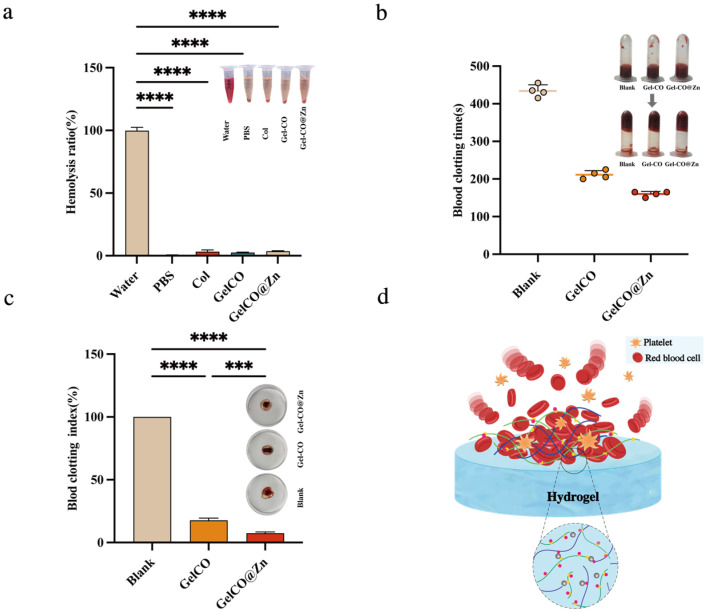
Hemostatic properties in vitro: (**a**) the hemolysis ratio of each group, the vertical bars represent the standard errors (*n* = 3); (**b**) whole blood clotting time, the vertical bars represent the standard errors (*n* = 4); (**c**) blood clotting index value for each group, the vertical bars represent the standard errors (*n* = 4); and (**d**) schematic illustration describing the hemostatic effects of the hydrogel (*** *p* < 0.001, **** *p* < 0.0001).

Hemolysis performance is an indispensable index to evaluate the safety of biological materials. In general, the smaller the hemolysis rate, the better the blood compatibility [47]. Therefore, the biocompatibility of the hydrogels was first assessed by hemolysis test. When Gel–CO and Gel–CO@Zn were incubated with blood cells, it was found that the hemolysis rates of both hydrogels were lower than 5%, with no significant difference compared to the negative control group. Moreover, it can be seen from the test tube photos that only the liquid of the positive control (water) was bright red, and the test tubes were light yellow, which was similar to the negative control (Figure 5a). Consequently, Gel–CO and Gel–CO@Zn hydrogels had good hemocompatibility. Subsequently, blood clot formation time and hemostatic performance of Gel–CO and Gel–CO@Zn hydrogels were evaluated by the blood clotting assay. It was found that the clotting time of recalcified mouse whole blood in the control group was approximately 433 ± 16.52 s (Figure 5b). In contrast, the Gel–CO (211.3 ± 11.09 s) and Gel–CO@Zn (160 ± 7.07 s) groups demonstrated significantly shorter clotting times. We speculated that bullfrog collagen contained in the hydrogels might help to promote blood coagulation.

Blood clotting index (BCI), another widely utilized quantitative indicator, evaluates a material’s capacity to promote blood coagulation, where a lower BCI value correlates with enhanced clotting activity [48]. Therefore, the hemostatic efficacy of hydrogels was further quantified through BCI analysis. As shown in Figure 5c, the Gel–CO@Zn hydrogel significantly exhibited lower BCI values (7.47 ± 0.95%) compared to the Gel–CO (17.63 ± 1.86%) hydrogel or the blank. Furthermore, the incorporation of Zn^2+^ in Gel–CO@Zn was associated with decreased BCI values, which was consistent with the results of a reduction in clotting time. Therefore, we hypothesized that the interaction of blood components with Zn^2+^ in Gel–CO@Zn could promote platelet activation. In addition, the stable interactions formed within the porous structure of Gel–CO@Zn enhanced both the rate and efficiency of clot formation (Figure 5d).

#### 3.5.2. In Vivo Hemostatic Evaluation of Hydrogels by Mice Liver Injury and Tail Amputation Models

**Figure 6:**
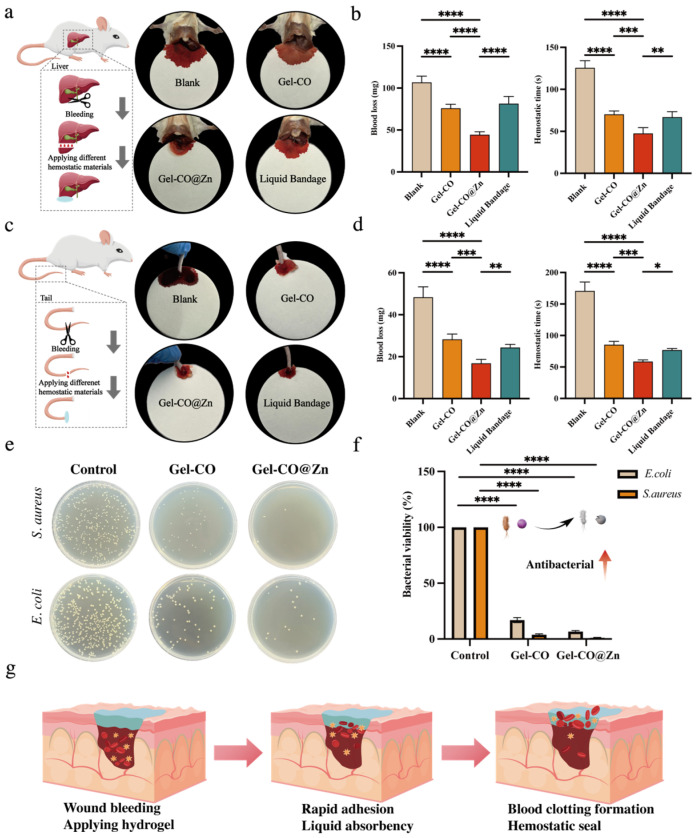
(**a**) Schematic diagram and photographs of liver resection: (**b**) blood loss and hemostasis after trans-hepatic resection (*n* = 5); (**c**) schematic diagram and photographs of tail hemorrhage in different treatment groups; (**d**) blood loss and hemostasis time of trans-tail truncation (*n* = 5); (**e**) analysis of the antibacterial function of the Col-based hydrogels; (**f**) bacteria viability % of *S. aureus* and *E. coli*. (*n* = 3); (**g**) the mechanism of the designed Col-based hydrogels for accelerating hemostasis (* *p* < 0.05, ** *p* < 0.01, *** *p* < 0.001, and **** *p* < 0.0001).

Hemostatic materials are needed in cases where bleeding is out of control, such as surgical or trauma situations [49]. The hydrogels showed good adhesion, self-healing ability, and procoagulant activity in vitro. To evaluate the in vivo hemostatic performance of two hydrogels, we constructed models of tail amputation and hemorrhaging liver in Kunming mice to simulate the bleeding from a human wound. As shown in Figure 6a, the liver lobes of male Kunming mice were first cut open with scissors to cause them to bleed. The hydrogels were immediately applied to the bleeding site, using a commercial liquid bandage as a positive control group. It was found that after the application of the hydrogels, the blood loss volume of liver injury in mice was significantly reduced from 106.7 mg to 75.76 mg for Gel–CO and to 44.26 mg for Gel–CO@Zn (*p* < 0.0001) by comparison with the blank group (Figure 6b). At the same time, the hemostasis time of the Gel–CO and Gel–CO@Zn groups of the liver injury site was also reduced from 125.6 s to 70.2 s and 47.4 s, respectively (*p* < 0.0001). In contrast, the hemostasis time of commercial hemostatic agents was 66.80 s, and the blood loss volume was 81.34 mg. Therefore, Gel–CO@Zn demonstrates superior hemostatic efficacy compared to commercial hemostatic agents. A similar hemostasis effect was also found in mice tail vein bleeding models (Figure 6c,d). Compared to the blank, Gel–CO@Zn reduced the blood loss volume from 48.34 mg to 16.78 mg (*p* < 0.0001) and hemostasis time from 170.6 s to 58.4 s (*p* < 0.0001). Whereas the hemostasis time of the commercial hemostatic agent was 76.80 s, and the blood loss volume was 24.34 mg. Our results showed that the Gel–CO@Zn hydrogel had the highest hemostasis potential of all other groups. The hemostatic mechanism of Gel–CO@Zn is illustrated in Figure 6g. When applied to a bleeding wound, Gel–CO@Zn hydrogel could rapidly adhere to the wound, forming a physical barrier that effectively prevents blood from leaking out. In addition, the Gel–CO@Zn hydrogel contains a variety of hemostatic compounds such as collagen, aldehyde groups, and Zn^2+^, which could help to activate platelets and promote blood cell adhesion and hemostatic film formation.

Considering the frequent coexistence of hemorrhage and microbial contamination in traumatic injuries, we further evaluated the antimicrobial efficacy to validate the multifunctional capacity of this hydrogel system. The antibacterial activity of Gel–CO sponge and Gel–CO@Zn were evaluated using *S. aureus* and *E. coli.* Colony-forming unit (CFU) assays revealed that two bacterial growths were significantly inhibited when incubated with the dilution in Gel–CO and Gel–CO@Zn for 18 h at 37 °C (Figure 6e). Quantitative analysis results are shown in Figure 6f. Compared to the control group, the bacterial viability of the Gel–CO@Zn group reduced to 1.06 ± 0.32% for *S. aureus* and 6.73 ± 0.80% for *E. coli*. Notably, Gel–CO without Zn^2+^ incorporation also showed significant antibacterial activity. The bacterial viability results were as follows: 3.81 ± 0.87% for *S. aureus*; 16.9 ± 2.26% for *E. coli*. The antibacterial properties of biomaterials can protect wound tissue from external bacterial infections [50]. Consequently, the Gel–CO@Zn hydrogel with good antibacterial properties has more advantages in hemostatic applications, such as the development of clinical hemostatic biomaterials.

## 4. Conclusions

In this study, we established the extraction method of type I collagen from bullfrog skin using citric acid, achieving a high-purity product with an extraction yield of 36.28%. Based on extracted bullfrog skin collagen combined with oxidized hyaluronic acid, we developed a novel Gel–CO@Zn hydrogel with a double crosslinking system of Schiff base bonding and Zn^2+^ coordination. The Gel–CO@Zn hydrogel exhibited enhanced mechanical properties, strong tissue adhesion (16.58 kPa), rapid self-healing (<6 h), low hemolysis (<5%), and effective antibacterial activity against *S. aureus* (1.06% survival) and *E. coli* (6.73% survival). In vivo studies demonstrated that Gel–CO@Zn significantly reduced blood clotting time and blood loss in mouse models. The developed Gel–CO@Zn hydrogel has promising applications, such as emergency hemostasis, surgery, and infection control. However, to become a hemostatic product, the Gel–CO@Zn hydrogel still needs to be validated using animal models that are closer to human clinical conditions. In addition, evaluation of long-term stability is also necessary to ensure clinical safety. From the perspective of sustainable development, this low-cost and multifunctional new biomedical material based on the collagen of bullfrog skin not only provides new ideas for the high-value utilization of bullfrog skin but also contributes to reducing environmental pollution and the sustainable development of the bullfrog industry.

## Data Availability

The original contributions presented in the study are included in the article. Further inquiries can be directed to the corresponding author.

## Abbreviations

Col	Collagen
OHA	Oxidized hyaluronic acid
HA	Hyaluronic acid
BCI	Blood clotting index
CMCS	Carboxymethyl chitosan
